# Comparison of rapid tests for detection of rifampicin-resistant *Mycobacterium tuberculosis *in Kampala, Uganda

**DOI:** 10.1186/1471-2334-9-139

**Published:** 2009-08-26

**Authors:** Sam Ogwang, Benon B Asiimwe, Hamidou Traore, Francis Mumbowa, Alphonse Okwera, Kathleen D Eisenach, Susan Kayes, Edward C Jones-López, Ruth McNerney, William Worodria, Irene Ayakaka, Roy D Mugerwa, Peter G Smith, Jerrold Ellner, Moses L Joloba

**Affiliations:** 1 Joint Clinical Research Centre, Kampala, Uganda; 2 Department of Medical Microbiology, Makerere University College of Health Sciences, Kampala, Uganda; 3 Department of Infectious and Tropical Diseases, London School of Hygiene & Tropical Medicine, London, UK; 4 Department of Medicine, Makerere University Medical School, Kampala, Uganda; 5 University of Arkansas for Medical Sciences, Little Rock, AR, USA; 6 Uganda-Case Western Reserve University Research Collaboration, Kampala, Uganda; 7 Department of Medicine, New Jersey Medical School – University of Medicine and Dentistry of New Jersey (UMDNJ), Newark, NJ, USA; 8 Makerere University-UMDNJ Research Collaboration, Kampala, Uganda

## Abstract

**Background:**

Drug resistant tuberculosis (TB) is a growing concern worldwide. Rapid detection of resistance expedites appropriate intervention to control the disease. Several technologies have recently been reported to detect rifampicin resistant *Mycobacterium tuberculosis *directly in sputum samples. These include phenotypic culture based methods, tests for gene mutations and tests based on bacteriophage replication. The aim of the present study was to assess the feasibility of implementing technology for rapid detection of rifampicin resistance in a high disease burden setting in Africa.

**Methods:**

Sputum specimens from re-treatment TB patients presenting to the Mulago Hospital National TB Treatment Centre in Kampala, Uganda, were examined by conventional methods and simultaneously used in one of the four direct susceptibility tests, namely direct BACTEC 460, Etest, "in-house" phage test, and INNO- Rif.TB. The reference method was the BACTEC 460 indirect culture drug susceptibility testing. Test performance, cost and turn around times were assessed.

**Results:**

In comparison with indirect BACTEC 460, the respective sensitivities and specificities for detecting rifampicin resistance were 100% and 100% for direct BACTEC and the Etest, 94% and 95% for the phage test, and 87% and 87% for the Inno-LiPA assay. Turn around times ranged from an average of 3 days for the INNO-LiPA and phage tests, 8 days for the direct BACTEC 460 and 20 days for the Etest. All methods were faster than the indirect BACTEC 460 which had a mean turn around time of 24 days. The cost per test, including labour ranged from $18.60 to $41.92 (USD).

**Conclusion:**

All four rapid technologies were shown capable of detecting rifampicin resistance directly from sputum. The LiPA proved rapid, but was the most expensive. It was noted, however, that the LiPA test allows sterilization of samples prior to testing thereby reducing the risk of accidental laboratory transmission. In contrast the Etest was low cost, but slow and would be of limited assistance when treating patients. The phage test was the least reproducible test studied with failure rate of 27%. The test preferred by the laboratory personnel, direct BACTEC 460, requires further study to determine its accuracy in real-time treatment decisions in Uganda.

## Background

Developing countries account for 95% of active tuberculosis (TB) cases and deaths due to TB worldwide [1,2]. In developing countries, many national TB control programs have low case detection rates and once a case is detected, cure may also be difficult because of poor case holding, high default rates and insufficient control of drug prescription [3]. In such a setting, an added threat lies with the potential for development and spread of multi drug-resistant tuberculosis (MDR-TB), defined as resistance to at least isoniazid and rifampicin, as well as extensively drug-resistant tuberculosis (XDR -TB) caused by MDR strains resistant to any fluoroquinolone and at least one of three injectable second-line drugs (i.e., amikacin, kanamycin, or capreomycin) [4,5]. Although the DOTS strategy is effective in the management of drug susceptible TB, outbreaks of MDR-TB and XDR-TB strains that are untreatable by the DOTS program threaten to complicate TB control [6-8]. In places where drug susceptibility testing is not routinely performed patients with MDR-TB are likely to become re-treatment cases and will be managed using a standardized regimen, such as 2 months of streptomycin [S], isoniazid [H], rifampicin [R], pyrazinamide [Z] and ethambutol [E] followed by 1 month of H, R, Z, E and 5 months of H, R, E. As the majority of re-treatment cases have susceptible TB and will respond to the simpler standard regimen (2HRZE/6HE) this practice is expensive for the program and also exposes the majority of re-treatment cases to unnecessary drug toxicity. For those with MDR-TB, the re-treatment regimen has a risk of adding a single drug to a failing regimen, which leads to worsening resistance profile and outcomes [9]. Previous studies have indicated that delayed initiation of appropriate treatment for MDR-TB is associated with increased mortality and spread of the MDR strains [10-13]. Typically in developing countries, if susceptibility testing is done, results are not available for 3–4 months. To improve patient survival, reduce unnecessary costs and toxicity during re-treatment, and minimize transmission of MDR-TB strains, a rapid method for detection of MDR-TB is a high priority for developing countries.

Uganda has a high burden of tuberculosis with an estimated prevalence of 561 cases per 100,000 of the population [2]. The prevalence of MDR-TB in new cases of TB has been reported to be low at less than 2% [2,14]; however, we have recently reported that 12.7% of re-treatment cases attending the National Tuberculosis and Leprosy Program (NTLP) clinic in Kampala had MDR-TB [9]. Although drug susceptibility testing is not routinely undertaken in Uganda it would be advantageous to know the drug susceptibility status of re-treatment patients to facilitate appropriate patient management. As part of a larger project to investigate strategies for controlling MDR-TB in re-treatment cases in Kampala we undertook an assessment of new technologies for rapid detection of MDR-TB. In this population resistance to rifampicin may be considered a good predictor of MDR-TB where less than 0.05% of re-treatment patients were found to have mono resistance to the drug [9]. There are several competing technologies that have been proposed for rapid detection of resistance directly from the sputum of tuberculosis patients. We have investigated four different technologies to assess their suitability for implementation in a TB reference diagnostic laboratory in Africa for the rapid assessment of resistance to rifampicin in smear positive re-treatment patients. The purpose of the study was to compare the feasibility of the technologies prior to selecting one for implementation in a larger study on the impact of new interventions to control MDR-TB in Kampala. The four technologies investigated were liquid culture (BACTEC 460, Becton Dickinson), an in-house bacteriophage based test, a Line Probe Assay (INNO-LiPA Rif.TB, Innogenetics) and drug impregnated strips on solid culture (E-Test, AB Biodisk). Performance characteristics and costs were compared using the indirect BACTEC 460 drug susceptibility test on culture as the reference standard.

## Methods

### Study setting

The culture-based methods were carried out in the TB laboratory of the Joint Clinical Research Centre (JCRC) in Kampala, while molecular assays were performed in the Department of Medical Microbiology at Makerere University. The JCRC TB laboratory supports clinical studies as well as routine patient care. On average, each month the JCRC TB laboratory receives 600 sputum specimens, for either diagnosis or follow-up. The Department of Medical Microbiology performs both applied and basic TB research and works closely with the JCRC laboratory.

### Study population and Ethics

The study population comprised adult (≥ 18 years) TB cases who presented to the Uganda NTLP clinic at Mulago Hospital in Kampala, Uganda during the period March 2004 to December 2005. Patients with smear positive disease who had been previously treated for TB and received at least two months of therapy were invited to join the study. All of the patients were admitted to the hospital ward to initiate therapy, which included daily injections of streptomycin. Subjects were classified according to reason for re-treatment, i.e., treatment failure, relapse, or defaulted treatment based on a previous treatment card or chart review. Sputum specimens were collected for culture and sensitivity testing, sampling was consecutive and not randomized. This being a feasibility study results from the rapid methods under evaluation were not used for patient management and only drug susceptibility results from the indirect testing were reported to the clinic.

The study protocol was approved by the AIDS research sub-committee of the Uganda National Council of Science and Technology together with the Institutional Review Boards at the University of Medicine and Dentistry of New Jersey and London School of Hygiene & Tropical Medicine and Hygiene.

### Study design

Each of the rapid tests was evaluated using sputum specimens collected from re-treatment patients with smear positive tuberculosis. In addition to testing by one of the rapid methods specimens were assessed by smear microscopy and cultured in the BACTEC 460. Indirect drug susceptibility testing was performed on isolates obtained. Technicians performing the rapid tests were kept blind to the results of the indirect drug susceptibility tests until the final comparative analysis. For the purposes of this feasibility study, the rapid tests were evaluated consecutively rather than concurrently, with the test under scrutiny being run alongside the existing routine activities of the laboratory. A maximum of five specimens were tested each week. As this study was to gauge the feasibility of integrating the various technologies into a routine laboratory, each test was assessed for a period of at least three months.

### Laboratory procedures

Early morning spot sputum specimens were collected in sterile disposable 50 ml polypropylene centrifuge tubes and taken to the laboratory. Specimens delivered to the lab after 2 pm, were refrigerated at 2 – 8°C and processed the following morning. Specimens (2.5 to 10 ml) were decontaminated using a final concentration of 1% NaOH and concentrated at 3000 × g for 15 minutes. The sediment was reconstituted to 2.5 ml with phosphate buffer pH 6.8, and used to prepare smears and cultures on Middlebrook 7H10 agar and in BACTEC 12B. Smear microscopy was performed using auramine and graded according to ATS/CDC guidelines [15]. Only smear-positive specimens were selected for subsequent testing by one of the rapid methods.

Growth from 12B vials was used for indirect drug susceptibility testing in the BACTEC 460, according to the manufactures' instruction [16]. Quality control for the indirect BACTEC drug susceptibility test included weekly monitoring with H37Rv, a pan-susceptible strain, according to the guidelines of the Clinical and Laboratory Standards Institute (CLSI; formerly the National Committee for Clinical laboratory Standards). In addition, new lots of BACTEC 12 B medium and new drug lots were controlled with drug resistant strains. External quality assessment included participation in the quality assurance program for drug susceptibility testing administered through the WHO/IUATLD Supranational Laboratory Network. Confirmation of *M. tuberculosis *complex was determined by either the BACTEC NAP test (Becton Dickinson) or PCR for IS *6110 *[17]. Following sampling for microscopy and culture, part of the remaining inoculum was used for the rapid direct test under investigation. The direct tests were set up at the same time as the routine cultures, with the exception of the LiPA where samples were batched.

To set up the direct BACTEC susceptibility test, 0.1 mL of stock rifampicin solution (80 μg/ml) was added to a 12B vial, the same final concentration (2.0 μg/ml) as used with the indirect method. This vial was then inoculated with 0.1 mL of 1:10 dilution of the digested and decontaminated sputum sediment. A second BACTEC 12B vial, without rifampicin, was prepared in the same manner to serve as a growth control. Vials were pre-incubated before testing on the BACTEC 460 instrument, depending on the degree of smear positivity. 1+ and 2+ smears were held 1 day, and 3+ and 4+ smears were held 2 days before testing commenced [16]. Vials were read daily until a control Growth Index (GI) of at least 20 was reached. Susceptibility to rifampicin was determined by comparing the change in GI values between the rifampicin-containing vial and the control vial. When the GI in the drug vial decreased in relation to the GI of the control vial, the organism was considered "susceptible". Conversely, when the GI of the drug vial increased in relation to the GI of the control vial, the organism was considered "resistant". Indirect testing confirmed the direct susceptibility results.

For the INNO-LiPA Rif.TB test, the sputum sediment was heated to 80°C for 60 minutes to lyse the MTB. To improve lysis, samples were sonicated for 10 minutes. DNA in the lysate was amplified in a PCR targeting the *rpoB *gene where mutations associated with rifampicin resistance are located. Subsequently, the amplicon was hybridized to immobilized-membrane bound probes covering overlapping wild-type sequences [18,19]. The interpretation of the results was done in accordance with the manufacturer's instructions (Innogenetics, Inc, Ghent, Belgium).

The direct phage assay was set up according to the method described by McNerney, et al. [20,21]. Briefly, 1 ml of the inoculum (direct sediment or 1:10 dilution) was added to 1 ml of 7H9 broth containing rifampicin (final concentration 4 μg/ml). A second drug-free control tube was also similarly set up. The tubes were pre-incubated at 37°C for 24 hours. Then 50 μl of D29 phages was added to each tube for a final concentration of 8 × 10^6^/ml. The tubes were incubated for 90 minutes at 37°C to allow the phages to infect the mycobacteria cells. 0.25 ml of 100 mM ferrous ammonium sulphate was added to neutralize extracellular phages. The mixture was added to 1 – 2 ml of stationary phase grown *Mycobacterium smegmatis mc*^*2 *^155, in a tube to which 9 ml of molten LB agar (1.5%) was added. The mixture was poured to a 90 mm Petri dish. The plates were sealed and incubated at 37°C for 16 hours. The plates were examined for plaques and the number of plaques recorded. The presence of plaques in the drug-free sample and no plaques in the rifampicin-containing sample indicated that the MTB in the sputum was susceptible to rifampicin. However, if plaques were seen in both samples, the MTB was considered rifampicin resistant.

For direct Etest, the manufacturer's directions (AB Biodisk, Solna, Sweden) were followed with the exception of using the sputum sediment in place of a mycobacteria suspension [22,23]. In order to reduce contamination, two 7H10 plates were made selective by addition of Polymixin B (200 IU/ml), Amphotericin B (10 μg/ml), Carbenicillin (50 μg/ml) and Trimethoprim (20 μg/ml); and each plate was then inoculated with 0.3 ml of sediment, swabbing evenly over the entire plate. Plates were pre-incubated for 48 hours at 37°C in a CO_2 _incubator, after which a rifampicin Etest strip was added to one plate. This procedural modification was necessary to obtain good growth on the plates with the Etest strip. The second plate without an Etest strip was used as a growth control. Plates were sealed and read weekly, until an elliptical zone of inhibition was observed. A critical concentration of 1.0 μg/ml, similar to that used for agar dilution in Middlebrook, was used as a break point for determining rifampicin resistance.

### Statistical analysis

The statistical significance of the differences in the sensitivities and the specificities of the tests evaluated were assessed using Fisher's Exact test for comparing binomial proportions.

### Cost per test

Cost analyses, in US dollars, were performed for the four direct rapid methods and the indirect BACTEC 460 drug susceptibility test. Overall expenses were determined by calculating the direct costs associated with each test, including reagents, consumables and labor. The cost of preventive maintenance of equipment and general operating supplies, such as gloves, gowns, masks, disinfectants were included. Since sputum specimens needed to be decontaminated, buffered and concentrated before being tested, routine specimen processing costs were included in the overall cost assessment. Since smear-positive samples were required for testing, the cost for microscopy was also included. Labor costs were based on remuneration of laboratory personnel for the time in hours spent undertaking the task. General overheads and items of a site specific nature such as manager and director salaries, clerical wages, capital equipment outlay, and facility operational expenses were excluded. Such costs vary widely among Ugandan laboratories as well as throughout the rest of Africa.

### Turn around times

Turn around time (TAT) was estimated as the time, in days, from the receipt of specimens to the time results were available in the laboratory. For the LiPA test, estimation of the turn around times commenced from when a batch of samples, stored at -20°C, was retrieved for analysis.

## Results

A total of 203 smear-positive specimens yielding MTB isolates were tested. Eighty percent of the specimens had 3+ or 4+ smears (17, 23, 50 and 113 for 1+, 2+, 3+ and 4+ graded smears, respectively). The distribution of specimens tested by the direct methods was 39 BACTEC, 44 Etest, 77 phage and 43 LiPA. Out of 203 specimens examined, 17 primary BACTEC cultures had no growth, thus indirect drug susceptibility results were not available for comparison with the direct test results. Additionally, 27 direct tests were not interpretable due to various reasons: i) growth failures occurred in one direct BACTEC, one Etest, and seven phage; ii) control failures occurred in one direct BACTEC and eight phage; iii) two contaminated cultures in each of the direct BACTEC, Etest, and phage; and iv) three PCR failures in the LiPA. With the direct tests, failures were observed throughout the smear positivity range, and were not associated with only specimens containing low numbers of MTB.

The ability of each direct test and the indirect BACTEC drug susceptibility test to yield interpretable results is shown in Table 1. Direct testing by BACTEC, Etest, phage or LiPA yielded a result in 87, 89, 73, and 86% of samples tested, respectively. The LiPA was the only direct test to provide a higher number of test results than the indirect test (37 vs. 33). The phage method performed least well with a success rate of 73% compared to 96% for the indirect method.

**Table 1:** Comparison of various direct and the indirect BACTEC 460 susceptibility methods according to AFB smear grade.

*Smear* *Grade*	*Direct BACTEC* *(n = 39)*			*Direct E-test * *(n = 44)*			*Direct phage* *(n = 77)*			*Direct LiPA* *(n = 43)*		
	No. Tested	Direct Available	Indirect Available	No. Tested	Direct Available	Indirect Available	No. Tested	Direct Available	Indirect Available	No. Tested	Direct Available	Indirect Available
**1+**	3	1 (33%)	1 (33%)	2	0	1 (50%)	2	0 (0%)	1 (50%)	10	7 (70%)	2 (20%)
**2+**	6	6 (100%)	6 (100%)	4	4 (100%)	4 (100%)	9	4 (44%)	6 (67%)	4	3 (75%)	3 (75%)
**3+**	7	5 (71%)	7 (100%)	17	15 (88%)	16 (45%)	19	14 (74%)	18 (95%)	7	7 (100%)	7 (100%)
**4+**	23	22 (96%)	23 (100%)	21	20 (95%)	21 (100%)	47	38 (81%)	47 (100%)	22	20 (91%)	21 (95%)

**TOTAL**	**39**	**34** **(87%)**	**37** **(95%)**	**44**	**39** **(89%)**	**42** **(96%)**	**77**	**56** **(73%)**	**74** **(96%)**	**43**	**37** **(86%)**	**33** **(77%)**

### Performance of direct tests

Comparable results between the direct methods and the indirect BACTEC were available for 159 (78%) of the 203 specimens comprising 34, 44, 56 and 30 samples tested by direct BACTEC, Etest, phage and LiPA respectively. Overall, of the 159 isolates tested in the indirect BACTEC, 115 (72%) were rifampicin susceptible and 44 (28%) resistant. Performance characteristics of the four methods are shown in Table 2. Using the indirect BACTEC as the gold standard, the sensitivity and specificity for detecting rifampicin resistance in direct BACTEC was 100% and 100%, for direct E-test 100% and 100%, direct phage 94% and 95% and for direct LiPA 87% and 87%. Overall concordance with the indirect BACTEC for determining rifampicin susceptibility and resistance ranged from 100% (direct BACTEC and Etest) to 93% (LiPA). However, it should be noted that none of the differences in sensitivity and specificity observed were statistically significant.

**Table 2:** Performance of rapid test methods for detecting rifampicin resistance compared to indirect BACTEC.

	Indirect BACTEC			
	
		Resistant	Susceptible	Total
**Direct** **BACTEC**	Resistant	4	0	4
	
	Susceptible	0	30	30
	
	Total	4	30	34

**Direct** **Etest**	Resistant	9	0	9
	
	Susceptible	0	30	30
	
	Total	9	30	39

**Direct** **Phage**	Resistant	15	2	17
	
	Susceptible	1	38	39
	
	Total	16	40	56

**Direct** **LiPA**	Resistant	13	2	15
	
	Susceptible	2	13	15
	
	Total	15	15	34

### Test costs

Total costs for each rapid method and the indirect BACTEC susceptibility test are shown in Table 3. The distribution of costs attributed to each of the rapid tests by reagents/consumables and labor are indicated. The indirect BACTEC and LiPA had similar total costs and were the most expensive. Total costs for the Etest and phage assay were similar and the least expensive. In-house preparation of media for these tests kept test-specific reagent costs low. The direct BACTEC cost was in mid-range. Since testing a sample by LiPA required two extra control strips, testing in batches produced substantial costs savings.

**Table 3:** Cost comparison (US$) of indirect BACTEC and four rapid methods

	Indirect BACTEC	Direct BACTEC	Direct Etest	Direct Phage	Direct LiPA
**Reagents and Consumables:**					
Sputum Processing	1.01	1.01	1.01	1.01	1.01
Fluorescent Microscopy	1.33	1.33	1.33	1.33	1.33
Test-specific	24.44	16.64	6.99	5.63	30.60
Overhead*	3.23	3.23	3.23	3.23	3.55
**Total**	**30.01**	**22.21**	**12.56**	**11.20**	**36.49**
					
**Labor:**					
Sputum Processing	0.56	0.56	0.56	0.56	0.56
Fluorescent Microscopy	1.04	1.04	1.04	1.04	1.04
Test-specific	8.23	2.99	2.62	4.78	1.83
Overhead	1.82	1.82	1.82	1.82	2.00
**Total**	**11.65**	**6.41**	**6.04**	**8.20**	**5.43**
					
**Total Cost per Specimen**	**41.66**	**28.62**	**18.60**	**19.40**	**41.92**

* Overhead included preventive maintenance of equipment and general operating supplies, such as gloves, gowns, masks and disinfectants.

### Turn around times (*TAT*)

The LiPA and phage results were available in 2–4 days, being the most rapid of the four tests. The average time to a positive direct BACTEC was eight days. The Etest had the longest TAT of 14–37 days (mean, 20), which was still faster than the conventional indirect BACTEC with time to positive ranging from 8–60 days (mean, 24). The TAT generally coincided with degree of smear positivity. Direct BACTEC results were available as early as four days with all 4+ specimens and one 3+ specimen. The remaining 3+ and half of the 2+ specimens had direct BACTEC results within seven days. The other half of 2+ specimens took longer, up to 20 days. The direct BACTEC result for the one analyzable 1+ specimen was available in 6 days.

## Discussion

The current conventional methods for determining drug susceptibility are based on testing isolates rather than direct testing of organisms in patient specimens. To provide susceptibility data in a clinical and public health relevant time frame, results should be available within a few days of specimen collection. Molecular and phage tests, both commercial and those developed in-house may provide results in 1–4 days. Such examples are the INNO-LiPA tested in this study, HAIN Rif.TB (HAIN Lifescience GmbH, Nehren, Germany) [24,25], molecular beacon assay [26], FASTPlaqueTB-RIF (Biotec Laboratories Ltd., Ipswich, UK) [27], and in-house phage test [28]. Methods that provide results in around 8–12 days rely on culture and use some means of detecting growth before it is macroscopically evident. These include BACTEC 460 radiometric TB system [16], BACTEC MGIT 960[29], Etest [22,23], nitrate reductase assay (NRA) [30], microscopic observation drug susceptibility (MODS) assay [31], and other microplate assays using oxidation-reduction indicators [32] for early detection of growth. All of these are promising methods for rapid detection of rifampicin and isoniazid resistant MTB and could potentially be set up to test second-line drugs, which would facilitate rapid detection of XDR-TB. Most of these methods have previously been used with AFB smear-positive specimens; but few evaluations have been done in high disease burden settings such as Uganda. Since our interest was in examining the feasibility of introducing one these technologies into a a TB reference diagnostic laboratory we trialed each of the tests independently on a limited number of samples each week in order to reflect the caseload of re-treatment patients attending the clinic.

The LiPA met the qualifications of providing results very quickly and results could have been available even sooner if they had not been batch tested. This method yielded more interpretable results than the indirect culture based method when applied to the same samples (Table 1). Our results are not surprising in that this test looks for mutations in the genome and does not require viable bacilli. Other authors have reported the LiPA used directly with sputum specimens detects MTB in specimens missed by culture [33]. The lower proportion of evaluable specimens using the BACTEC culture in this comparison may have been due to non-viable MTB in patients on drug therapy or killing of bacilli during the NaOH decontamination process. Three of the PCR reactions for the LiPA failed, possibly due to inhibition. It is probable that the yield of positive LiPA results would be increased if a more rigorous DNA extraction method were used instead of crude lysates. However, this would increase the hands on time required and overall cost of the test. When compared to the indirect direct susceptibility results, there were four discrepant LiPA results (Table 2). Two of these (resistant by indirect BACTEC, susceptible by LiPA) may be due mutations outside the "hotspot" region in the *rpoB *gene which are not covered by the LiPA test. Two discrepancies in which the LiPA was resistant and the indirect test was susceptible are unexplainable, but could be due to a mixture of resistant and susceptible strains in one sample. All four LiPA discrepancies where confirmed with at least one other indirect BACTEC test. Our results suggest that this test could be used to triage patients for appropriate TB treatment regimen and control measures. Commercially available line probe assays were recently endorsed by WHO for this purpose, subject to the availability of appropriate training and supervision. In addition to speed, the test has a second advantage over culture based methods in that samples can be treated to render them non- infectious. The major disadvantage of the LiPA compared to the other technologies we investigated is the high cost and requirement for separate clean room facilities to avoid contamination of the amplification reaction.

The in-house phage test was also ultra-rapid. Unfortunately, many of the tests did not yield results (Table 1). Some of the reasons for this were microbiological contamination and failure of *Mycobacterium smegmatis *indicator plates or unexpected control results. Three discrepant drug susceptibility results were observed; one R indirect BACTEC but S phage; and two S indirect BACTEC but R phage. The one discrepant isolate which was sensitive by the phage test but resistant by indirect BACTEC was resistant by repeat indirect BACTEC and LiPA susceptibility tests. However, the isolate remained sensitive by repeat phage test suggesting phage failure to detect resistance. The two discrepant isolates that were resistant by phage but susceptible by indirect BACTEC were susceptible by repeat indirect phage as well as BACTEC. The major advantage of the direct phage is its low cost. However, in our experience it was technically difficult to adapt, perform and interpret when compared to the other tests.

The direct BACTEC was found to be fairly rapid, technically easy and fitted well in the work flow of the laboratory. For this reason it was the preferred test of the laboratory staff. Although most of the specimens tested were from the multi-bacillary patients, the sensitivity and specificity of direct BACTEC of 100% is encouraging. In principle, the test could be easily extended to other liquid culture systems such as the Mycobacteria Growth Indicator Tube (MGIT) BACTEC 960 (Becton Dickinson, Towson, MD),) and BacT/Alert (bioMérieux, Hazelwood, MO) and recent work by El-Sayed et al [34] supports this. It has the added advantage that the technology may also be used for diagnosis and, if required, testing susceptibility to other anti-tuberculosis drugs. The laboratory is currently undertaking further studies with liquid culture including the direct detection of resistance to isoniazid and rifampicin using MGIT 960.

The direct Etest also had 100% agreement with the indirect BACTEC. It was simple to set up, learn, and perform and is inexpensive. We determined that the rifampicin susceptible isolates had MICs in the range of 0.002 to 1.0 μg/ml and all resistant isolates had a MIC >256 μg/ml, an added benefit of the Etest. However, TATs were much longer than for the other tests suggesting the Etest would be of limited value for triaging patients.

We believe this is the first report comparing head-to-head these four technologies for detecting drug resistance from sputum in a TB endemic country with automated liquid culture. It should be noted that our evaluation was based on samples with a high percentage of heavy smear-positive specimens, more specimens in the 1+ to 2+ range need to be tested to determine the sensitivity of these methods with low numbers of organisms. Similarly, the lack of statistical significance of the test performance data reflects the small sample size. A larger study would be required to fully determine the sensitivity, specificity and predictive values of the tests in this setting.

An important consideration when testing *M. tuberculosis *in the laboratory is safety as great care must be taken to avoid exposure to infected aerosols. Indirect culture based methods require handling of large numbers of bacilli and stringent safety precautions are necessary, including negative pressure rooms and microbiological safety cabinets. Direct testing of sputum involves much lower numbers of bacilli reducing the risk to laboratory personnel. However, inhalation of a very small number of bacilli can result in infection and tuberculosis disease and the direct BACTEC, Etest and phage all require protective facilities to microbiological safety category level III. The risk of aerosol production and accidental exposure increases each time viable bacteria are handled. The BACTEC test requires less manipulation of open samples than either the Etest or the Phage assay as so might be considered the least risky procedure. As previously mentioned the LiPA may be considered the safest technology due to the opportunity to sterilize samples prior to testing, when they may be handled in a standard laboratory without fear of infection.

The majority of diagnostic laboratories in TB endemic countries are poorly resourced and do not have the capacity to undertake culture of *M. tuberculosis*, and drug susceptibility testing in such settings is currently restricted to a few central referral laboratories. Until recently these reference laboratories were restricted to culture using homemade Lowenstein Jensen slopes, a slow and cumbersome procedure. However, a recent change in international policy and the launch of the Global Laboratory Strengthening Initiative by the STOP-TB partnership has changed the outlook for TB laboratories. A program of refurbishment funded by international donors has led to the introduction of automated liquid culture systems. This has coincided with an expansion of activities to treat and control MDR-TB. The tests we have investigated would be appropriate for implementation in the reference laboratories of developing countries where treatment for MDR-TB is available. Ideally new tests should be robust, fast and efficient. Our finding that the direct BACTEC method was the most convenient in this a TB reference laboratory suggests that further evaluation of automatic liquid culture methods is warranted. The LiPA test was rapid and has advantages of safety. However, the necessity to avoid contamination of the PCR requires dedicated clean room facilities. Investment will also be needed to retrain technical staff in molecular skills and this option may be less attractive for some laboratories.

There are concerns that resistance to rifampicin may not be a reliable marker for MDR-TB in some settings, especially where rifampicin mono-resistant isolates are common. Review of indirect BACTEC drug susceptibility data from the laboratory during 2000–2006 showed that 164 (90%) of the 181 rifampicin resistant isolates were also isoniazid resistant. However, there may be clinical situations in which rapid detection of isoniazid resistance, in the absence of rifampicin resistance, may be useful to construct an effective treatment regimen. Thus, inclusion of isoniazid in rapid tests may be desirable. Furthermore, with concern about possible emergence of XDR-TB in Africa, adding testing for resistance to second-line drugs (one fluoroquinolone and two aminoglycosides) to the rapid test is logical. The rapid culture-based methods can easily be adapted to test for isoniazid resistance and additional drugs. Several of the molecular techniques, Genotype MTBDR (Hain Lifescience) and molecular beacon assay, already test for the *katG *and *inhA *gene mutations that are associated with isoniazid resistance. It is likely that the molecular assays can be easily modified to include gene targets for second-line drugs. However, the sensitivity of such tests has yet to be determined and preliminary reports suggest they may be less accurate than tests for rifampicin.

## Conclusion

A high correlation was observed between the indirect rifampicin susceptibility results using the BACTEC 460 and those from the four rapid methods when applied to smear-positive sputum specimens. Although the number of specimens tested was small, the results demonstrate that personnel in this lab setting can be trained to successfully use a number of different test platforms. The test preferred by laboratory personnel, the direct BACTEC, was neither the fastest nor the cheapest but was preferred on grounds of convenience. The line probe assay was considered the safest test and was rapid but was expensive and required separate laboratory facilities to avoid PCR contamination. We recommend that further investigation be undertaken to determine the efficacy and cost effectiveness of direct liquid culture and the commercial line probe assay for detection of MDR-TB in an sub-Sahara African setting. We suggest that the delays in obtaining results from the direct Etest and the lack of robustness and inconvenience of the in-house phage based test preclude their application for detection of drug resistant tuberculosis in a clinical setting.

## Competing interests

The authors declare that they have no competing interests.

## Authors' contributions

SO performed the phage assays and participated in drafting the manuscript.

HT assisted with the phage assays and training for LiPA.

BBA performed the LiPA assays and participated in drafting the manuscript.

FM performed the Bactec and E-tests.

SK participated in general supervision of laboratory work, collection of cost data and drafting the manuscript.

KDE, EJ, RM, PGS, RDM, JE and MLJ participated in the conception and design of the study, general supervision of the research, and critical revision of the manuscript.

AO, IA and WW: participated as study physicians.

All authors read and approved the final version of the manuscript.

## Pre-publication history

The pre-publication history for this paper can be accessed here:

http://www.biomedcentral.com/1471-2334/9/139/prepub
